# Archeological Frauds

**Published:** 1883-07

**Authors:** F. W. Putnam

**Affiliations:** Cambridge


### ARCHEOLOGICAL FRAUDS.

As an illustration of the demand and supply of archeological material, I will call attention to a carved stone representing a naked child about two feet in length, which was said to have been dug up near the Hot Springs in Arkansas. The carving was partly enclosed by a cement, which, it was said, covered the stone when it was found. This was received at the Peabody museum, with its history, apparently well authenticated, describing it as an antique. This piece of carving proved to be a child of the 'Cardiff giant' family. The fraud was unquestionable; and the image was returned to its owner with a full statement of the evidence against it, and the request that in the interest of science the object should be destroyed. Since then I have heard nothing more of it, and in case it has not been destroyed this notice will serve to put others on their guard. This is, however, but one of the many fraudulent specimens offered for sale ; and we have received a number of pipes, tubes, dishes, ceremonial and other objects, made in Philadelphia, and sold as having been found in such or such a locality. The variety of these articles made by the Philadelphia manufacturer, and the character of the work are such that many have found their way into collections in this country, and not a few have supplied the foreign demand for American antiquities. A manufacturer in Indiana confines his attention chiefly to 'mound-builders' pipes,' which are carved from stone, and offered in a systematic method to collectors. In Ohio a large business has been done in the so-called gorgets, cut from blue slate, and in hematite celts. In southern Illinois, a few years ago, many specimens of pottery were made, until the demand fell off so that one manufacturer acknowledged that he was no longer paid for his trouble by their sale. Another man who made this pottery is, I believe, no longer living; but much of his work is still extant. This list might be lengthened; but it is already sufficient to show that the demand for 'antiquities' is considerable in this country, and that we are not behind the old world in keeping up the supply. F. W. PUTNAM, in *Science.*

CAMBRIDGE, Feb. 19.

